# Longevity

**Published:** 1875-06

**Authors:** 


Longevity.—Active exercise of the brain is not unfavorable to good health or long life. Mental training, a well-balanced mind, a cheerful, contented disposition, and temperate habits, are, with rare exceptions, found indispensible to longevity. These presuppose a harmonious development of the whole body, and particularly of all parts of the brain. The world's hardest workers and noblest benefactors have usually been long-lived. The brain-working classes—clergymen, lawyers, physicians, merchants, scientific men, and men of letters—live very much longer than the muscle-working classes; the greatest and hardest brain-workers of history have lived longer on an average than brain-workers of ordinary ability and industry; clergymen are longer lived than any other great class of brain-workers. Of a carefully prepared list of five hundred of the greatest men in history, the average age was upwards of 64. The average age at the present time of all classes of persons who live over twenty years is about 50. The greatest men of the world, accordingly, have lived longer on the average than men of ordinary ability in the different occupations, by fourteen years; six years longer than physicians and lawyers; nineteen or twenty years longer than mechanics and day-laborers; from two to three years longer than farmers; and a fraction of a year longer than clergymen, who are the longest-lived classes in our modern society. Any chronology comprising from one to five hundred of the most eminent personages in history, at any cycle, will furnish an average longevity of from 64 to 70 years.

A Healthy Brain.—A healthy brain should show, by the outward signs of clear, easily working intelligence, well-balanced faculties, and commanding will, that its several organs, if such there be, or its several modes of action, if it works as a whole, are properly developed and adjusted by themselves and in relation to each other. We are, of course, applying the term healthy to the brain, as signifying much more than freedom from disease. But there can be no sound working brain without enough good blood to build it, repair it, and furnish the materials for those molecular changes which are the conditions essential to all nervous actions, intellectual and volitional, as well as those of lower grade. No good blood without a proper amount of proper food and air to furnish materials, and healthy organs to reduce a sufficient quantity of these materials to a state fit to enter the circulation. No healthy organs, strictly speaking, except from healthy parents, and developed and maintained by proper stimuli, nourishment, and use. All these, except healthy parents, nearly all of us have it in our power to secure by right living. As for healthy parents, we can at least see to it that our own offspring are provided with these, through temperance and purity on our part.

---

Nurses should be cheerful. A tearful and melancholy countenance has in itself a depressing effect, and a steady, cheerful temper of mind is almost as important a requisite in a nurse, as tenderness and affection.

---

A crust is hard fare, but none at all is harder.

